# Limitations of current copyright frameworks for large language models trained on scientific literature

**DOI:** 10.3389/frai.2026.1781692

**Published:** 2026-04-10

**Authors:** Yuanyuan Huang, Hui Liu

**Affiliations:** Institute of Medical Information, Chinese Academy of Medical Sciences & Peking Union Medical College, Beijing, China

**Keywords:** copyright law, fair use, large language models (LLMs), scientific literature, transformative use

## Abstract

Recent copyright lawsuits against artificial intelligence (AI) and large language model (LLM) developers have ignited debates over how to balance technological innovation with the public interest. In the scientific research field, the performance and reliability of LLMs trained on scientific literature (SciLit-LLMs) depend heavily on access to comprehensive, up-to-date full-text sources. This paper argues that the current copyright framework, including the U.S. fair use doctrine, often regarded as a flexible solution for AI-related copyright issues, is ill-suited for SciLit-LLMs. First, the normative values emphasized in scientific—such as accuracy, transparency and interpretability—fundamentally conflict with the “transformative use” requirement central to copyright law. Second, the expression of scientific literature is intended to ensure scientific precision rather than to convey creative originality, remains insufficiently considered under current copyright law. Third, the fair use doctrine’s emphasis on limiting the proportion of use from a single copyrighted work contradicts the need for comprehensive training on information-dense scientific texts. Finally, commercial use restrictions impede the sustainable development of SciLit-LLMs and preclude a mutually beneficial model for researchers, publishers, developers, and the public. Imposing current copyright restrictions on these models is unjustified, unnecessary, and risks perpetuating scientific biases. We therefore propose reconstructing copyright exceptions for scientific literature and removing commercial use restrictions to better support scientific innovation.

## 1 Introduction

The rapid advancement of large language models (LLMs) and AI technologies has promoted automation and intelligent changes across various fields in recent years. The training corpora for early LLMs are primarily sourced from books, newspapers, and online content. However, a recent study predicts that publicly available text data will be fully exhausted by 2026–2032 (Villalobos et al., 2022). High-quality copyrighted works are expected to play an increasingly significant role in the future development of LLMs. However, using copyrighted materials for training LLMs has sparked lawsuits against leading AI developers, including OpenAI, Microsoft, Stability AI, Google, and NVIDIA (Milton et al., 2025). OpenAI acknowledges that “it would be impossible to train today’s leading AI models without using copyrighted materials” (OpenAI, 2023). Other industry leaders, including Stability AI and Microsoft, have maintained that “they should be allowed to use these copyrighted works without permission due to a furthering of research, and due to the fact that they are transforming the outputs” (Barnett, 2024). However, in high-profile AI training copyright infringement cases, the defeat of AI companies has forced AI researchers and developers to adopt greater caution in selecting training datasets (Meckes and Grasser, 2025).

Unlike models used in general or creative domains, scientific literature presents a distinct copyright character that warrants special consideration. This paper focuses on the scientific literature domain for three reasons. First, authors of scientific publications generally do not expect direct financial returns from their works; instead, they seek to enhance academic influence and professional reputation. Thus, this field confronts less tension that copyright law aims to balance. Second, scientific writing emphasizes factual and verifiable content rather than subjective or aesthetic expression, making it less in line with the creative originality requirement under copyright law. Third, the scientific domain demands a high degree of accuracy and transparency, which conflicts with the copyright doctrine of transformative use.

The hypothesis of this paper is that the current copyright framework is fundamentally ill-suited to LLMs trained on scientific literature (SciLit-LLMs). This paper proceeds in three stages. First, it provides a descriptive analysis of how current copyright frameworks function in practice and reveals their misalignment with the nature of scientific works. Second, it analyzes the potential risks applying these frameworks to SciLit-LLMs. Third, it advocates reforming copyright law to better align with the epistemic and communicative aims of science.

This study uses biomedical literature as a representative case. Biomedical research generates a vast, high-quality, and rapidly growing body of publications, making it an essential resource for LLM training. Its focus on accuracy, transparency, and reproducibility often conflicts with copyright restrictions, and its limited subjective or aesthetic expression further highlights the misfit between creativity-based copyright doctrines and scientific writing.

## 2 How current copyright frameworks operate in the context of scientific literature

### 2.1 Copyright exceptions in the context of research and text and data mining

#### 2.1.1 Text and data mining exceptions across jurisdictions

In recent years, many countries have supported using copyrighted work for AI pre-training. For example, the EU permits explicitly “text and data mining for the purposes of scientific research” (European Union, 2019). According to Article 30–4 of Japan’s Copyright Act, the use of copyrighted works for the purpose of data analysis is permissible only when it is conducted without the intention of enjoying the thoughts or sentiments expressed in the work. These developments reflect a global trend toward recognizing text and data mining as a legitimate and socially beneficial use of copyrighted materials, particularly in advancing scientific and technological innovation.

#### 2.1.2 Commercial vs. non-commercial use limitation

At present, most countries recognize copyright exceptions for non-commercial purposes. For example, the UK Government (2022) and the European Union (2019) have enacted regulations permitting the use of copyrighted materials for non-commercial scientific research for AI training. Meanwhile, a few countries make no explicit distinction between commercial and non-commercial AI training. For example, Japan Copyright research and information center (2020) and Singapore Statutes Online (2021) have adopted even more progressive policies, allowing the use of copyrighted data for both commercial and non-commercial AI training, positioning them as the most AI-friendly countries (Hays, 2024).

#### 2.1.3 Legal classification of scientific works under copyright law

Whether scientific literature is considered copyright-protected content is a controversial issue. For exvample, UK copyright law explicitly states that “copyright work” includes “original literary, dramatic, musical or artistic works,” and “sound recordings, films,” it does not include scientific works (Legislation.gov.uk, 1988). Japanese copyright law stipulates that copyrightable works are defined as “a creatively produced expression of thoughts or sentiments that falls within the literary, academic, artistic, or musical domain” (Japan Copyright research and information center, 2020). Scientific literature is not included. Unlike most other European countries, Germany and Spain classify scientific works as copyrightable (Esteve, 2024). Spanish commentators argue that “scientific discoveries, theories, methods, and ideas” within scientific works are not protected by copyright, while the “wording, images or figures created by the author to explain the content is copyright protected” (Esteve, 2024).

### 2.2 Reasons for applying the U.S. Fair use framework

In 1967, Article 9 (2) of the Berne Convention first explicitly introduced the “three-step test,” establishing a framework for copyright applicable to the 181 signatory countries. The three criteria are as follows: “permit the reproduction of such works in certain special cases,” “such reproduction does not conflict with a normal exploitation of the work,” and “does not unreasonably prejudice the legitimate interests of the author” (Legal Information Institute, 1971). The “special cases” identified in the first criterion allow member countries the flexibility to define the scope of exceptions according to their national legal traditions. This test allows national legislatures to define specific exceptions, but it remains a relatively static legislative model in which unlisted uses are typically not exempted.

The fair use doctrine in U.S. copyright law is widely regarded as compatible with the three-step test outlined in the Berne Convention. Countries such as the Philippines, Israel, and South Korea have also incorporated fair use provisions into their national copyright laws (Geiger et al., 2014). The fundamental distinction between the three-step test and fair use lies in their regulatory approaches: the three-step test requires legislatures to specify in advance the conditions under which copyright exceptions apply, whereas fair use grants judicial institutions the discretion to balance all relevant interests on a case-by-case basis. In practice, however, national copyright regimes show less divergence than might seem. Even in jurisdictions such as the EU, the UK, and Japan, where text-and-data-mining or non-commercial research exceptions are recognized, such uses cannot conflict with the normal exploitation of the work and cannot unreasonably prejudice the author’s legitimate interests (European Union, 2019; Japan Copyright research and information center, 2020). More broadly, whenever domestic laws have copyright exceptions, these must be formulated within the normative boundaries of the Berne Convention and remain subject to its second and third-step constraints, both of which are inherently reflected in the logic of U.S. fair use. Consequently, the U.S. fair use doctrine is adopted as the primary analytical benchmark, as it offers the most comprehensive, judicially tested, and analytically coherent framework for understanding how the Berne Convention’s normative rationale operates in practice, particularly in addressing AI training challenges.

### 2.3 Limits of fair use in the context of SciLit-LLMs

#### 2.3.1 Purpose and character of the use

The central consideration of this factor lies in two core criteria: the commercial and transformative nature of the use. Among them, judicial precedents have clearly demonstrated that “transformative use” has become the most significant consideration (Purdue Global Law School, 2021; Brooke, 2023), even though non-commercial uses generally have stronger fair use claims, they are not decisive. Section 107 of the US Copyright Law explicitly identifies “teaching,” “scholarship,” and “research” as examples of non-commercial situations (U.S. Copyright Office, 2025), while the the transformative use is defined as “those that add something new, with a further purpose or different character and do not substitute for the original use of the work” (U.S. Copyright Office, 2025). According to Japanese copyright law, “It is permissible to exploit a work, in any way and to the extent considered necessary… in any other case in which it is not a person’s purpose to personally enjoy or cause another person to enjoy the thoughts or sentiments expressed in that work” (Japan Copyright research and information center, 2020). In other words, works protected by copyright are originally intended to enable individuals to experience or appreciate the thoughts or emotions conveyed by the creator.

In fair use cases on AI training, courts have reached differing conclusions under the first factor. In 2023, the landmark *Andy Warhol Foundation for the Visual Arts, Inc. v. Goldsmith* case held that the Warhol’s Prince series did not constitute fair use, as the original and secondary works served the same or highly similar purposes and the use was commercial (Andy Warhol Foundation, 2023). Although many scholars argue that AI training is insufficiently transformative (Shen, 2024), the landmark *Bartz v. Anthropic* held that using books to train a generative AI was “highly transformative,” since the outputs did not replicate the plaintiffs’ works and the training pursued a distinct purpose from the originals (Gollwitzer, 2025). The copyrighted books were creative works valued for reader enjoyment, while the AI’s purpose was generating summaries, stories, or code. In contrast, scientific papers convey objective knowledge, not artistic expression. Since their function generally aligns with that of SciLit-LLMs, such uses are typically not transformative.

Transformative requirements may also conflict with the models’ functional goals. If an LLM merely absorbs scientific papers and reproduces their content on demand, such use clearly lacks transformative character (Gervais et al., 2024). Allowing LLMs to retain memory can help mitigate hallucinations (Cyphert, 2024). Balancing accuracy and creativity is a challenge for general models, and their priorities should vary by context. While transformative use encourages models to rewrite original expressions or generate new content, SciLit-LLMs prioritize authenticity, accuracy, and transparency (Department of Health and Social Care of UK Government, 2019; World Health Organization, 2021), making them more likely to deviate from transformative use.

The infringement risks of SciLit-LLMs under the current copyright framework may vary depending on the type of service they provide. For example, N-gram is one of the earliest natural language processing techniques. It has been applied to scientific texts for tasks such as sentence auto-completion, spell checking, and grammar correction (Yazdani et al., 2019). With algorithmic advancements and the expansion of training data, the modern improved version, Infini-gram, has achieved high accuracy in next-word prediction and can reduce the perplexity of LLMs (Liu et al., 2024). Infini-gram can provide retrieval services over pre-training corpora or target databases without infringing copyright. A typical precedent is the *Authors Guild v. Google* case, in which Google collaborated with university libraries to scan book collections, allowing the users to view short “snippets” when searching for specific terms, but not the full content. The court ruled that Google’s use of the books was “transformative,” as it enhanced public knowledge without substituting the original works (Brooke, 2023). Similarly, we argue that if SciLit-LLMs only provide retrieval services—i.e., showing short “snippets” to help users quickly locate the articles they need while still requiring permission to access the full text—such use would also meet the standard of transformative use. However, SciLit-LLMs also integrate knowledge, automatically generate literature reviews, and answer questions that require deeper analysis and re-expression of original content. In such scenarios, LLMs do not change the purpose of using literature; instead, they achieve it more efficiently, which introduces greater uncertainty and controversy regarding copyright issues.

#### 2.3.2 Nature of the copyrighted work

This factor primarily examines “the degree to which the work that was used related to copyright’s purpose of encouraging creative expression” (U.S. Copyright Office, 2025). The Trade-Related Aspects of Intellectual Property Rights (TRIPS) states, “Copyright protection shall extend to expressions and not to ideas, procedures, methods of operation or mathematical concepts as such” (World Trade Organization, 1994). In the recent case of *Thomson Reuters v. Ross Intelligence Inc.*, although the court ruled that Ross’s use constituted copyright infringement, it also acknowledged that the legal documents lacked creativity, which favored Ross (Meckes and Grasser, 2025). In October 2023, OpenAI defended its use of copyrighted materials, which qualifies as fair use by presenting that “[t]he factual metadata and fundamental information that AI models learn from training data are not protected by copyrighted law” (U.S. Copyright Office, 2023). Literary and artistic works featuring imaginative, creative, and personalized expressions are less likely to qualify for fair use than those presenting facts.

Even in the most rigorous scientific literature, some scholars may express their humor (Heard et al., 2023). There are significant differences in the degree of creative expression in academic literature across various disciplines. Generally, works in literature, philosophy, and history tend to feature distinct linguistic styles, exhibiting higher levels of creativity, while literature in biomedicine, natural sciences, and mathematics typically demonstrates lower levels of creative expression. Within a scientific literature, different sections may exhibit varying degrees of expressive creativity—data charts, statistical results, and other factual content lack creativity, while the review, introduction, and discussion sections may reveal the author’s expressive style.

Scientific literature presents objective experimental data and systematic reviews. It often contains specialized terminology and definitions, with structures and formats that adhere to standardized guidelines, lacking the personalized expressions and imaginations in general creative works. Nevertheless, scientific literature is not reduced to numbers, graphs, and tables; instead, it is mainly presented in the form of text, which is primarily due to the complexity of facts that require understanding within a specific context. For example, a study investigating whether anemia is a risk factor for dementia in patients with chronic kidney disease (Koyama et al., 2023). In this article, understanding the findings cannot rely solely on statistical data; it also requires information on inclusion and exclusion criteria, variable definitions, testing methods, statistical models, result interpretations, and research limitations (Koyama et al., 2023). Scientific knowledge and facts must be understood within a context rich in expressive content, making it challenging to separate pure facts from expression.

Nonetheless, the purpose of expression in scientific literature fundamentally differs from that of literary and artistic works: it aims to convey facts with precision, rather than to reflect personal style or creative intent. Although copyright laws establish “creative expression” as a standard for protection, they do not list scientific literature as a copyright exception. As a result, the use of literature behind paywalls by SciLit-LLM does not fundamentally violate the spirit and purpose of copyright law. Yet, it may still be a legal infringement under the current legal framework. While the factual nature of a work may support a fair use defense, it carries less weight than factors such as transformative use or market impact, and is rarely sufficient on its own to preclude a finding of infringement.

#### 2.3.3 Amount and substantiality of the portion used of the copyrighted work as a whole

This factor seeks to reduce the proportion of training data derived from a single source of copyrighted content for LLMs. However, achieving this goal is nearly unattainable for SciLit-LLMs. Scientific literature is inherently information-dense, encompassing extensive details, data, and specialized terminology with highly relevant context. Consequently, this factor contradicts efforts to enhance the performance and accuracy of LLMs trained in this field.

#### 2.3.4 Effect of the use upon the potential market for or value of the copyrighted work

Among the four fair use factors, the first and fourth are the most decisive (Sag, 2024). The first factor considers the purpose and character of the use, including its commercial nature. The fourth factor evaluates the actual market impact, which is more decisive in nature but largely depends on the copyright holder’s ability to demonstrate market harm (Kessel, 2025). When copyright holders fail to provide evidence of market harm, courts may consider the commercial purpose as an indicator of whether the use is likely to serve as a substitute for the original market. The first three fair use factors are closely interconnected with the fourth factor. For instance, non-commercial use typically indicates a lower market impact. Second, using factual works is less likely to impact the market value of the original. Third, the smaller the proportion and less significant the content used relative to the entire work, the lower the potential for market substitution. Some countries have designated research use as an exception under copyright law; however, such use must still comply with the third step of the Three-Step Test—namely, that it does not unreasonably prejudice the legitimate interests of the author. Copyright law is designed to “achieve a fair balance between the rights and interests of authors and other rightsholders, on the one hand, and of users on the other” (European Union, 2019). Unauthorized use of literary and artistic works (such as novels, music, and films) for enjoyment often directly reduces their market demand, thereby undermining the economic interests of authors and rights holders. When these works are used solely for scientific research or educational purposes, these uses represent a relatively small portion of the overall market and generally do not significantly impact sales. This is the primary reason copyright law supports including education and research as exceptions to copyright restrictions.

In *Kadrey v. Meta*, the courts held that the use constituted fair use. Although the LLM was transformative, the decisive factor remained its market impact (Gollwitzer, 2025). The court found no evidence that the model’s outputs harmed the original market for the copyrighted works (Gollwitzer, 2025). However, the situation differs for SciLit-LLMs. Publishers of academic journals derive most of their copyright revenue from subscriptions by doctors, researchers, and academic institutions, making the scientific and educational sectors their primary markets. If researchers can obtain detailed insights from relevant literature through LLMs, their dependence on paywalled articles may decline. Even when LLMs rephrase or reorganize content without producing highly similar or duplicated text, the extraction of key information may reduce the need to access paywalled originals. Since the copyright holders of paywalled literature are typically publishers, pre-training with such literature without authorization harms the commercial interests of the publishers. Thus, even non-commercial SciLit-LLMs can affect the original market. Conversely, a commercial use does not necessarily harm the existing market. A highly transformative use can still qualify as fair use if it creates a new market rather than replacing the original one. For example, SciLit-LLMs that share scientific knowledge with the public serve a different audience and function, adding new channels for communication and education without substantially replacing the existing scientific publishing market.

Moreover, under the second fair use factor, factual works are generally more likely to qualify for fair use. However, this factor is secondary to the fourth factor concerning market impact, which is the most decisive. Paradoxically, LLMs trained on factual content are often more likely to affect the original market, revealing an inherent tension in applying fair use to AI training. For instance, in *Thomson Reuters v. Ross Intelligence*, the court rejected Ross’s fair use defense, finding that its use of copyrighted legal materials to train an AI research tool was commercial, non-transformative, and likely to harm Thomson Reuters’s market (Meckes and Grasser, 2025). The ruling in *Thomson Reuters v. Ross Intelligence* differed from those in *Kadrey v. Meta* and *Bartz v. Anthropic*, showing that differences in the nature of copyrighted works can lead to different market impacts from AI training. For highly creative literary works, AI training is more likely to qualify as fair use due to its strong transformative nature and limited market substitution effect. In contrast, for factual works such as scientific and legal literature, the knowledge transmission function often overlaps with the objectives of model training, making it difficult to qualify as fair use.

### 2.4 Copyright in the output phase

While fair use primarily concerns how copyrighted materials are used in training, potential infringement issues may also emerge at the output stage when the model produces new content. AI systems absorb and encode expressive elements of copyrighted works during training, potentially producing outputs that reproduce the originals’ aesthetic or expressive features, thereby weaking claims of transformative use (Gervais et al., 2024; Charlesworth, 2025). Some scholars note that the primary legal risk arises at the model’s output stage, where copyrighted content may be reproduced (Rahman and Santacana, 2023). As creators may claim infringement when generated content closely resembles original works, academic research has increasingly focused on this issue and explored scientific methods to address related legal challenges (Gervais et al., 2024; Sag, 2024; Sharma et al., 2025; Chu et al., 2024). However, copyright constraints in the output phase may conflict with the scientific pursuit of accuracy and reliability. For example, the core mechanism of differential privacy is to add noise to the training data to reduce the identifiability of an individual’s data, thereby effectively protecting the privacy of individuals included in the dataset (Dwork and Roth, 2014). This method can be used to reduce the similarity between the generated content and the original training data, which may help mitigate the risk of infringement to some extent (Elkin-Koren et al., 2023). However, the design objectives of differential privacy may diverge from the core requirements of SciLit-LLMs—the transparency of training data and the traceability of model outputs. In addition, the introduction of noise through differential privacy may reduce the accuracy of the model’s output (Dyda et al., 2021). Although privacy protection and data transparency often conflict in developing LLMs (Liao and Wortman Vaughan, 2023), privacy concerns are less prominent in scientific literature. With appropriate support from copyright law, SciLit-LLMs can achieve data transparency and output accuracy without having to compromise between privacy protection and copyright risks.

## 3 What risks will result if no reform occurs

### 3.1 The training process of SciLit-LLMs

Pre-trained LLMs are built on the Transformer architecture and are designed for natural language processing and generation. They are primarily categorized into Bidirectional Encoder Representations from Transformer (BERT) and Generative Pre-training Transformer (GPT). BERT employs the Transformer’s encoder architecture and is pre-trained with two primary tasks: Masked Language Model and Next Sentence Prediction (Devlin et al., 2018). Its bidirectional nature enables it to capture both the left and right context for each word in a sequence, making it highly effective for tasks requiring deep contextual understanding (Devlin et al., 2018). For example, in the field of biomedical literature, BERT has been widely adapted and studied. Notable examples include BlueBERT (Peng et al., 2019), BioBERT (Lee et al., 2020), PubMedBERT (Gu et al., 2021), and Bioformer BERT (Fang et al., 2023).

GPT is built on the Transformer’s decoder architecture and employs Masked Self-Attention to predict the next word sequentially based on the left context, enabling it to generate coherent and contextually relevant text (Radford et al., 2018). Early applications of GPT to biomedical literature yielded relatively poor performance (Moradi et al., 2021), with models like PubMedBERT-base, BioBERT-large, and RoBERTa-large outperforming GPT-3 In-Context learning for biomedical information extraction tasks (Gutiérrez et al., 2022). However, as training data and model architectures have advanced, the advantages of GPT models have become evident. In 2022, BioGPT, trained on 15 million PubMed abstracts, achieved leading results across several biomedical NLP benchmarks (Luo et al., 2022). Later that year, Stanford University released BioMedLM, a GPT-2 style autoregressive model trained on PubMed abstracts and open-access full-text articles, which demonstrated strong performance in biomedical multiple-choice question answering tasks (Bolton et al., 2024).

These models are pre-trained from scratch on scientific literature to optimize their performance in specific tasks. The training process typically comprises three phases. The first stage involves constructing a domain-specific dataset and vocabulary. The second stage focuses on pre-training the model using the BERT or GPT architecture, enabling it to learn the semantics and sentence structures inherent in scientific texts. The final stage is fine-tuning, where the pre-trained model is further specialized and optimized for specific downstream tasks.

### 3.2 Risk of biased and uneven performance under insufficient training data

Grounded in the utilitarian foundation of U.S. copyright law (Didsbury and Zhu, 2025), some scholars argue that public interest factor, such as the significant social and economic benefits of LLMs and the practical difficulties of obtaining full authorization, should be a key consideration (Kessel, 2025; Didsbury and Zhu, 2025; Singh, 2025; Lightstone, 2024). The copyright law restricts data diversity and has been proven to be an important source of AI bias that has long been overlooked (Tepski, 2017). Such restrictions lead to data imbalance and knowledge bias (Sag, 2024). Parameters scaling represents a common strategy for augmenting model accuracy, yet it inherently escalates model complexity and computational overhead, compromising processing speed. Beyond a specific threshold, the marginal gains derived from parameter augmentation tend to diminish, and further increases may not yield substantial performance improvements relative to dataset expansion. For instance, Bioformer_16L_ achieves only 0.1% lower accuracy than PubMedBERT, despite having 60% fewer parameters, and operates 2–3 times faster than PubMedBERT (Fang et al., 2023). Maintaining a fixed model architecture while expanding the full-text training corpus typically improves accuracy and generalization capability while preserving computational efficiency for downstream tasks.

The PubMed database boasts over 30 million abstracts, and PubMed Central (PMC), containing 10 million full-text articles, provides a robust foundation for training LLMs. Training on scientific texts from scratch has shown clear advantages (Gu et al., 2021; Luo et al., 2022). A significant challenge in applying LLMs to the medical field is the critical need for accuracy (Thirunavukarasu et al., 2023). Given the high standards for accuracy in medicine, optimizing models by incorporating more training data is essential. The training corpus for LLMs is partially derived from OA full-text articles. In the medical field, there are significant disparities in the proportion of OA literature across disciplines and diseases (Breugelmans et al., 2018; Ay et al., 2022), which may lead to uneven performance of LLMs when addressing different tasks and diseases. Relying solely on OA literature for training materials risks insufficient knowledge coverage, resulting in biased outputs. Ensuring that the training data is diverse, comprehensive, and representative is crucial for enabling models to respond effectively to various medical scenarios and diseases.

While abstracts provide condensed content with reduced redundancy, enabling more efficient learning and mitigating overfitting risks, their lack of detailed explanations, contextual background, and nuanced information inherently limits model comprehension depth. LLMs trained on both abstracts and full-text articles may degrade performance in some functions due to the need to balance depth with efficiency. For example, in biomedical relation extraction using BioBERT, the version pre-trained solely on PMC full-texts or PubMed abstracts outperformed the version trained on both across more tests (Lee et al., 2020). Training on a more extensive and diverse set of full-text sources will enhance performance in scenarios requiring greater depth and higher accuracy. In question-answer tasks, BioBERT pre-trained on PMC full texts achieved higher strict accuracy scores across more tests than its counterpart pre-trained on PubMed abstracts. The abstract-trained model achieved higher lenient accuracy scores (Lee et al., 2020), suggesting that using a larger proportion of full-text data may further improve generalization.

Increasing the quantity of training data does not always yield benefits, particularly when the data quality is low. The quality of the training dataset often plays a more critical role in enhancing the performance of LLMs. Nevertheless, scientific literature generally adheres to higher quality standards, primarily featuring new findings or valuable insights. While training materials are sometimes sampled to improve efficiency, this approach is less suitable for scientific literature, as each piece holds irreplaceable value. Incorporating more full-text data is always beneficial.

LLMs amplify biases at a systemic level and make them more difficult to detect. LLMs hold tremendous potential in promoting equitable access to medical knowledge, but the systemic biases they may introduce cannot be overlooked. In the era before LLMs, researchers could exhibit individual-level biases in topic selection, analysis, and interpretation due to subjective judgment or objective limitations. However, these biases tended to be diverse and dispersed, which diluted the overall impact of any particular bias. Additionally, researchers typically reflected on the limitations of their studies in published articles, making such limitations relatively transparent and foreseeable. In contrast, biases learned by LLMs are often systemic, difficult to detect, and hard to trace. For example, AI models trained on historical healthcare data might wrongly classify individuals from socioeconomically disadvantaged backgrounds—who historically received insufficient care—as healthy and not in need of treatment (Cho, 2021). Once embedded in the model, these biases can influence academic research and policies on a much broader scale and in more subtle ways, making them particularly concerning.

### 3.3 Quality degradation risks from restricted full-text access

The full texts of copyrighted papers, particularly those from non-open-access journals, typically undergo rigorous peer review, ensuring higher quality. However, the rise of predatory journals, which prioritize profit over academic integrity, poses a significant challenge. These journals often charge authors high fees for article processing while bypassing stringent peer review standards. Many predatory journals focus on scientific research and are typically OA publications (Rupp et al., 2019), disseminating false or misleading conclusions (Grudniewicz et al., 2019). Relying solely on OA journals as training materials may lead to higher proportions of lower-quality content, thereby amplifying potential risks and producing inappropriate policies.

Another significant limitation in applying LLMs in the scientific domain is the lack of recency (Thirunavukarasu et al., 2023). Emerging research topics that have not yet reached definitive conclusions demand more attention from researchers. For example, while the PMC database contains medical records from the late 1700s to the present, SciLit-LLMs have a greater need for access to the most recent studies. Training on the latest literature is critical to ensure that models have up-to-date knowledge. In this context, a sufficient number of recently published full-text articles is critical.

### 3.4 Barriers to specialized scientific innovation under limited literature access

General-purpose scientific LLMs are well-suited for multi-task and multi-scenario applications, while sub-specialized scientific models also offer irreplaceable advantages. Notable examples include EyeGPT (Chen et al., 2024) and RareBERT (Prakash et al., 2021). In the scientific field, various subdisciplines exhibit significant heterogeneity, each characterized by its own terminology, knowledge structures, and methodologies. Therefore, for SciLit-LLMs, models trained on specific subfields may be better equipped to accurately capture the semantic features and knowledge patterns of those domains. However, the volume of literature in some specialized fields is limited. If LLMs cannot adequately learn from sufficient literature, their effectiveness and utility will be significantly constrained. The scientific field requires timely access to and analysis of literature, especially when related research surges. Traditional manual reading and analysis methods struggle to meet these needs. In this context, the value of LLMs becomes evident, while the accessibility of training data remains a critical bottleneck to their effectiveness.

### 3.5 Risk of structural knowledge inequality from linguistic and legal fragmentation

Currently, scientific literature is predominantly in English, leading to the exclusion of non-English literature from many systematic reviews (Rockliffe, 2022). Establishing a robust translation system and developing learning models based on English scientific literature could enhance knowledge sharing across diverse cultural and linguistic backgrounds. However, this requires non-English-speaking countries to relax copyright restrictions on LLM training. English journals are primarily published by companies in Europe and North America (such as Elsevier, Springer, Wiley, SAGE, IEEE), resulting in compliance standards for AI training data being shaped by the legal frameworks of these developed countries. If these countries adopt more lenient policies toward AI training, developers may prioritize English literature due to lower legal risks. Consequently, researchers may increasingly rely on LLMs, leading to a decline in the use of non-English literature and the systematic neglect of regional scientific discoveries. While the Berne Convention allows different countries to set flexible copyright exceptions, this flexibility does not support the development of SciLit-LLMs. A unified, cross-border copyright mechanism is essential to ensure fair inclusion of multilingual medical literature in AI training data.

### 3.6 Risk of data opacity

In recent years, an increasing number of laws and regulations have aimed to enhance datasets transparency. For example, the EU Artificial Intelligence Act requires providers of general-purpose AI models to “draw up and make publicly available a sufficiently detailed summary about the content used for training of the general-purpose AI model” (European Union, 2024). The California Generative AI Copyright Disclosure Act mandates disclosure of all copyrighted works used to build or modify training datasets (CONGRESS GOV, 2024). However, despitethese regulations, AI developers have generally become increasingly cautious about disclosing the datasets over the past few years (Andrijevic, 2024). Beyond concerns about commercial competition and data security, a major reason is that many datasets contain unauthorized copyrighted content, and publicly disclosing these datasets could lead to mass copyright infringement claims (Andrijevic, 2024). In literature, music, and arts, the protection of creators’ passion and the pursuit of making their work accessible to a broader public are inherently challenging to reconcile, and this very tension lies at the core of the balance that copyright law seeks to maintain.

Nevertheless, for SciLit-LLMs, this situation can be avoided. The publication of academic papers is not driven by profit but by promoting knowledge dissemination. Authors are primarily motivated to address scientific issues, advance their careers, and enhance their reputations; they generally welcome increased use of their works. In practice, in hybrid journals, if authors wish to make their articles OA, they need to pay an article processing charge to the publisher, a practice that is fundamentally different from other creative fields. With less tension concerning innovation and public interest, implementing transparency requirements under a reasonable copyright framework is unlikely to increase legal risks. Furthermore, ensuring data transparency and output traceability are shared core demands among users, and this approach can enhance user trust while providing SciLit-LLMs with a competitive advantage. Conversely, mechanically applying the restrictions of traditional copyright law to SciLit-LLMs would not only create unnecessary legal barriers but also weaken their data transparency and traceability.

## 4 Reconstructing the copyright framework for SciLit-LLMs

### 4.1 Scientific literature as a copyright exception with permissible commercial use

One view argues that licensing, rather than legal reform, could address the compliance issues of SciLit-LLMs. However, given the vast data requirement of LLMs, comprehensive licensing is impractical due to high transaction costs (Didsbury and Zhu, 2025). In the highly centralized copyright ecosystem currently dominated by a few large publishers, completely reliance on licensing places model developers at a disadvantage during negotiations. This could make innovation based on SciLit-LLMs the privilege of a few resource-rich institutions, raising knowledge access costs and create new informational barriers.

Another view holds that collective licensing bodies may serve as an alternative to legal reform (Gervais et al., 2024; Rosati, 2025). These organizations composed of creators that centrally manage copyrights, negotiate licenses, collect fees, and distribute revenues (Department for Science, innovation and technology, 2024). Since fully exempting AI training as fair use may promote innovation but weaken creative incentives, while case-by-case compensation is costly and inefficient, a centralized licensing and compensation system under administrative oversight could provide a fair and transparent solution (Shen, 2024). Some scholars suggest that such mechanisms can balance creators’ right and AI development needs without amending copyright law (Sag, 2024). However, academic researchers seldom receive direct financial returns from their publications. Although the legitimacy of publishers’ control has been questioned (Esteve, 2024; Tennant, 2020; Hagve, 2020), no effective alternative currently exists. Given that dissemination and citation, rather than profit, motivate participation in academic publishing, the scientific literature domain largely avoids the inherent tension between copyright protection and AI innovation seen in other creative industries. Thus, collective licensing models may have limited relevance here.

If scientific literature were recognized as a statutory copyright exception, one concern is that high-quality summaries generated by LLMs may replace publishers’ market role, causing economic loss. Under the EU’s current framework, “scientific purpose” is an exception, but commercial use is restricted, depriving developers of the opportunity to provide reasonable compensation through normal commercial activities. A more balanced approach is to legally define scientific literature as a copyright exception category, not confined to scientific purposes, permitting full-text training and commercial use under a compensation system. This approach aligns with the spirit of the three-step test and enhances the law’s adaptability to technological change.

Specifically, LLM developers can offer literature services to researchers, academic institutions, and medical organizations. They can use service fees to support model maintenance and continuous development, and pay reasonable remuneration to publishers through a revenue-sharing mechanism. This not only compensates for the potential losses of publishers but also encourages publishers to cooperate with AI developers. Researchers can acquire scientific knowledge more efficiently, and the scope of dissemination of authors’ achievements is also expanded accordingly. As a result, the model of knowledge acquisition changes from institutions directly purchasing the right of use from publishers to institutions obtaining knowledge through LLM services, and LLMs then obtaining the right of use from publishers. This structure does not infringe upon the rights of copyright holders and promotes the efficient and equitable dissemination of scientific knowledge.

### 4.2 Establishing public education as a copyright exception

The development and application of SciLit-LLMs in the field of public education facilitate the dissemination of scientific literature and expand its potential market capacity, thereby promoting knowledge equity and scientific literacy. Traditionally, the general public has had limited access to scientific literature. SciLit-LLMs can transform complex research findings into readable, understandable, and interactive content, creating a new market for popularized scientific knowledge that did not previously exist. In an era of information overload and widespread misinformation, public-oriented SciLit-LLMs can serve as important channels for providing the public with accurate and evidence-based scientific information.

At the same time, since the primary market for scientific literature remains focused on academic research rather than public education, this emerging market for public knowledge dissemination does not overlap with publishers’ main market, whose revenues mainly rely on institutional and scholarly subscriptions. Consequently, SciLit-LLMs exert only a limited impact on the economic interests of publishers. From both the perspective of transformative purpose and minimal market effect, this type of use represents a socially beneficial and relatively low-resistance pathway for reform under the current copyright framework.

## 5 Conclusion

Enhancing the accuracy of SciLit-LLMs relies on expanding the scale of the pre-training database and improving data quality. However, current copyright restrictions present a significant barrier to achieving this. We argue that the current framework of copyright law is ill-suited to scientific literature. First, the primary purpose of SciLit-LLMs is to deliver objective, accurate, source-traceable information, which is inherently incompatible with the legal standard of transformative use. Moreover, unlike other creative works, there is no direct correlation between whether SciLit-LLMs qualify as transformative use and their impact on the original market. Second, the factual content in scientific literature often requires interpretation within a rich context and is difficult to separate clearly from the expressive content. Nevertheless, such expressions serve to convey facts with precision, rather than demonstrating creativity or individuality, making them fundamentally different from creative works. The current copyright framework fails to consider this distinction, resulting in SciLit-LLMs potentially being deemed infringing when training on paywalled literature, even if their use does not violate the spirit of copyright law. Third, while fair use encourages limiting the proportion of any single copyrighted work used in LLM training, the high information density of scientific literature makes such a requirement impractical. Fourth, allowing the commercial use of SciLit-LLMs could create a win-win scenario for researchers, publishers, developers, and the public—a possibility currently constrained by the non-commercial copyright restriction. We advocate including scientific literature within the scope of copyright exceptions and removing commercial use restrictions, provided that such reform remains consistent with the three-step test under the Berne Convention.

While this study primarily draws on examples from biomedical sciences, its reasoning and analytical logic can be extended to other scientific disciplines, such as agronomy, mathematics, and physics. In these fields, knowledge and expression are generally more objective and formulaic. In contrast, literature in the social sciences and humanities often involves narrative and interpretation, making the boundary between factual and expressive content more ambiguous. Accordingly, the applicability of current copyright law requires further evaluation. Overall, our analysis provides a general orientation for copyright reform, aiming to inspire further interdisciplinary dialogue to better align the reform with SciLit-LLMs.

This article is primarily conceptual. Currently, we have not found any empirical research that directly compares the effects of copyright restrictions on the bias and market substitution of SciLit-LLMs. This is likely because no model has yet been trained on a complete corpus of full - text scientific literature. Once such models and datasets become available, future studies may empirically test and quantify some of the claims in this paper.

## Data Availability

The original contributions presented in the study are included in the article/supplementary material, further inquiries can be directed to the corresponding author.
